# Supplementary figures and images for: Dendropanax morbiferus leaf extract facilitates oligodendrocyte development
Source: R Soc Open Sci. 2019 Jun 26;6(6):190266. doi: 10.1098/rsos.190266 (PMC6599778; doi:10.1098/rsos.190266)

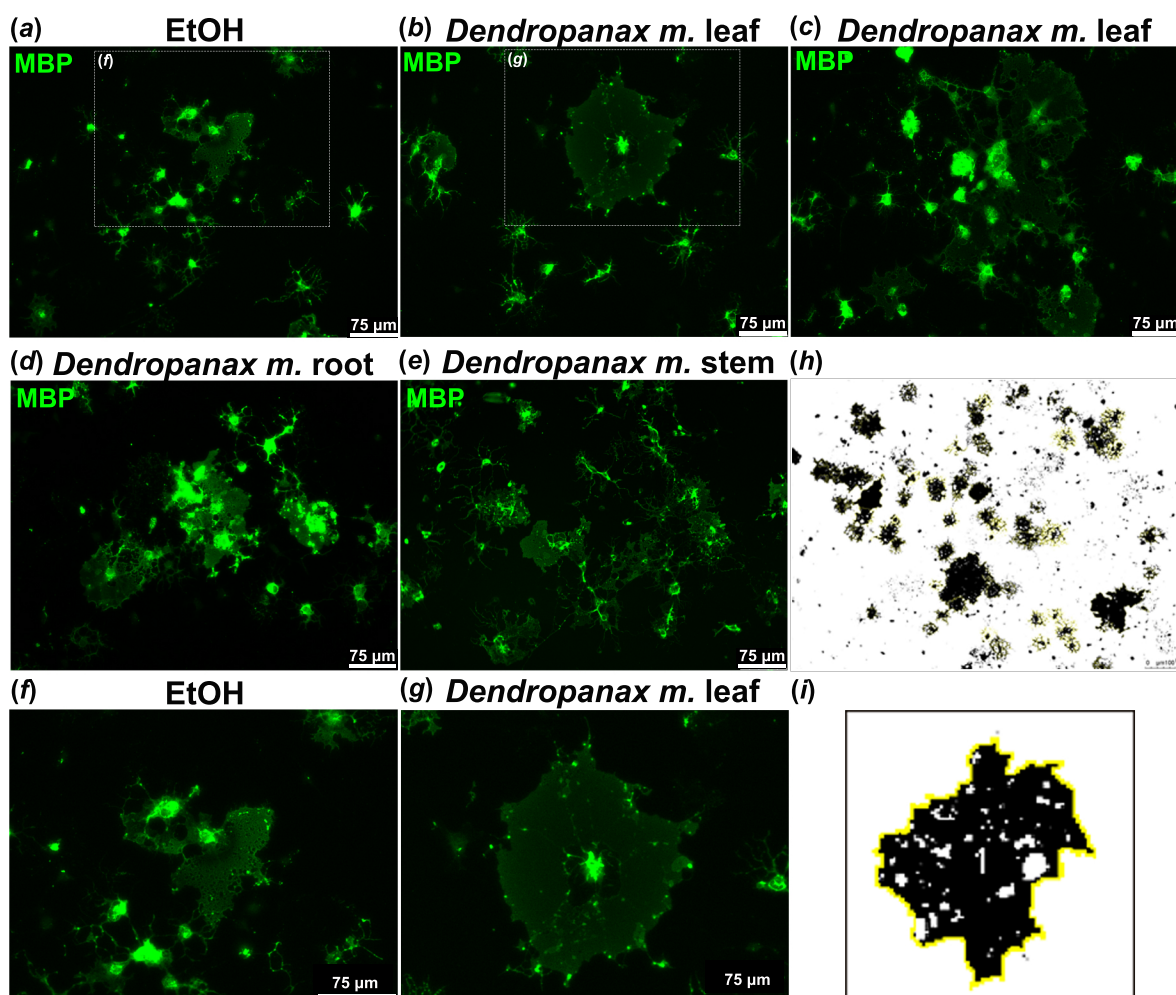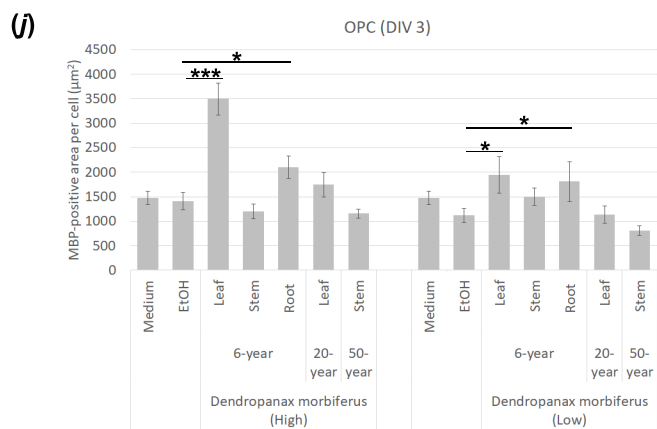

Supplement: The size of oligodendrocyte membrane sheath in the pure oligodendrocyte precursor cell cultures according to the plant part of the Dendropanax morbiferus [file rsos190266supp1.pdf]

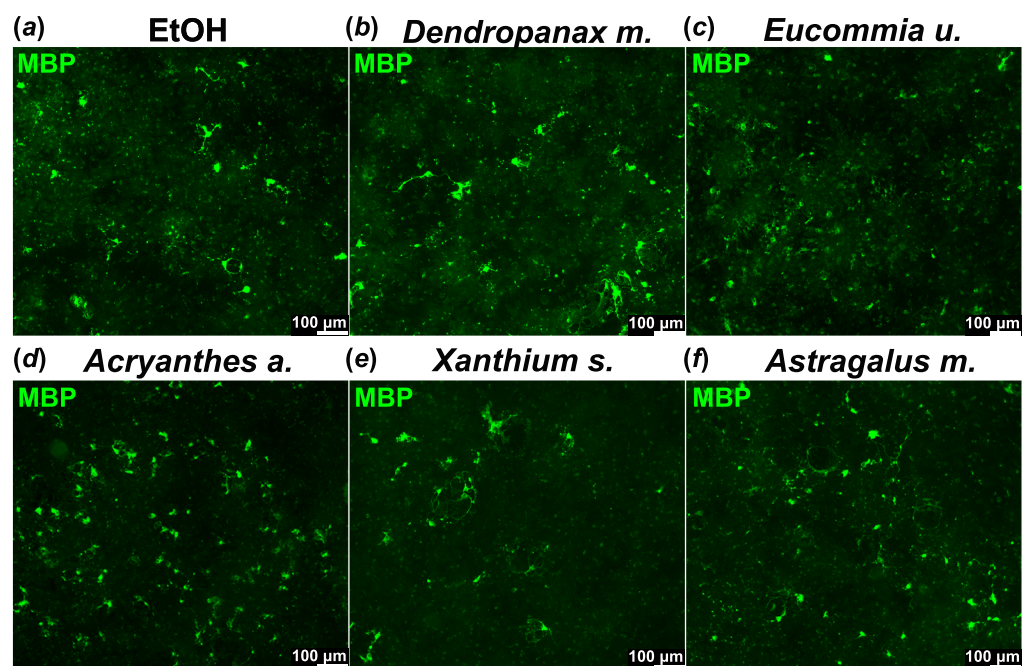

Supplement: The size of oligodendrocyte membrane sheath in the mixed glial culture according to the type of the medicinal plants. [file rsos190266supp2.pdf]

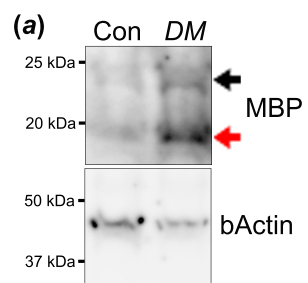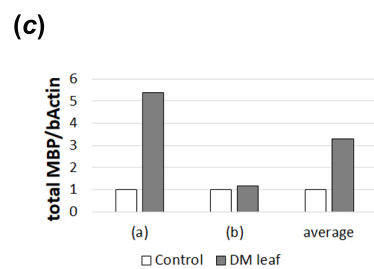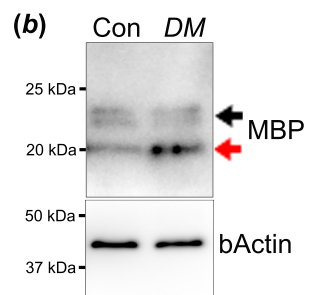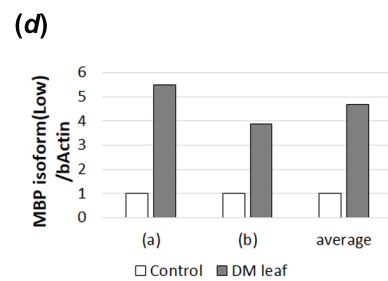

Supplement: Increased expression of MBP in the oligodendrocyte precursor cell cultures by incubation with the Dendropanax morbiferus extract [file rsos190266supp3.pdf]
